# Estimation of lung age via a spline method and its application in chronic respiratory diseases

**DOI:** 10.1038/s41533-022-00293-9

**Published:** 2022-09-29

**Authors:** Xiaolin Liang, Yanqing Xie, Yi Gao, Yumin Zhou, Wenhua Jian, Mei Jiang, Hongyu Wang, Jinping Zheng

**Affiliations:** 1 National Clinical Research Center for Respiratory Disease, State Key Laboratory of Respiratory Disease, Guangzhou Institute of Respiratory Health, First Affiliated Hospital of Guangzhou Medical University, Guangzhou, Guangdong China; 2 Firestone Institute for Respiratory Health, the Research Institute of St. Joe’s Hamilton, St. Joseph’s Healthcare; Department of Medicine, McMaster University, Hamilton, ON Canada

**Keywords:** Chronic obstructive pulmonary disease, Asthma

## Abstract

Lung age is a simplified concept that makes spirometry data easier to understand, but it is not widely used due to limitations in estimation methods. The aim of this study was to develop new equations to estimate lung age and to explore the application value of lung age in chronic respiratory diseases. Retrospective spirometric data of 18- to 80-year-old healthy subjects were used to develop the lung age estimation equations. Models were respectively built by multiple linear regression, piecewise linear regression, and the natural cubic spline method. Patients with chronic obstructive pulmonary disease (COPD) and asthma were subdivided into stages I–IV according to the severity of airflow limitation under the recommendation of the Global Initiative for Chronic Obstructive Lung Disease. Propensity score matching was performed to balance age, height and sex between healthy subjects and patients. The difference between lung age and chronological age (∆ lung age) of patients with COPD and asthma was analyzed. A total of 3409 healthy subjects, 280 patients with COPD and 285 patients with asthma data were included in the analysis. The lung age estimation equation with the best goodness of fit was built by the spline method and composed of FEV_1_, FEF_50%_, FEF_75%_ and height as explanatory variables. ∆ lung age progressively increased with the degree of airflow limitation in patients with COPD or asthma. Lung age estimation equations were developed by a spline modeling method. Lung age may be used in the assessment of chronic respiratory patients.

## Introduction

Chronic respiratory disease is a global health problem that brings heavy economic and health burdens to society^1^. The prevention and management of chronic respiratory diseases have become a great challenge. Spirometry is an important tool in the diagnosis, assessment, and management of chronic respiratory diseases^2,3^. However, the result of spirometry contains various parameters and may be complicated for some patients to understand. Spirometry-derived lung age (LA), a simplification of spirometry data, might be an alternative tool in the management of chronic respiratory diseases.

The concept of “lung age” was first proposed in 1985 to make spirometry data easier to understand and as a physiological instrument to evaluate the lung function damage caused by smoking^4^. A lung age older than the chronological age is considered an indication of the accelerated decline or impairment of lung function, and the difference between lung age and chronological age (i.e., ∆ lung age) is used to estimate the severity of this functional impairment. For example, if a 50-year-old man has a lung age of 60 years old, then his ∆ lung age is 10 years, indicating there may be an impairment of his lung function. A randomized controlled trial showed that telling smokers their lung age can improve the success rate of smoking cessation^5^. Lung age is also used as a clinical indicator in studies regarding physiological changes in individuals with morbid obesity^6^ and treatment efficacy in asthmatic patients^7^. However, it has not yet been widely used or fully exploited due to the doubt regarding the current lung age estimation methods^8^.

As a simplified lung function indicator, lung age was originally estimated by the backward calculation of the reference equation of spirometric parameter (usually the forced expiratory volume in 1 s [FEV_1_]) based on the assumption that the lung age of an individual equals the chronological age of a healthy nonsmoker who has the same FEV_1_ level as the individual (Morris et al.^4^, 1985; Hansen et al.^9^, 2010; Newbury et al.^10^, 2010). However, this estimation method may decrease the reliability of lung age, as it estimates lung age by using only one spirometric parameter^11^, whereas various spirometric parameters can reflect the functional changes of the lung during aging or in the context of disease. More importantly, lung age derived by this method is a mean value, not a normal range of the population. Neglecting the normal variability in lung function between individuals makes lung age unable to be correctly interpreted as normal or abnormal^8^. To overcome these problems, Yamaguchi et al.^11,12^ proposed the use of multiple linear regression to develop lung age estimation equations, with the chronological age of healthy nonsmokers as the dependent variable and spirometric parameters as well as height as explanatory variables. The limitation of this method is the assumption that the relationship between age and spirometric parameters can be approximated by a linear function^11^. Previous studies^13^ have revealed that the relationship between age and spirometric parameters is nonlinear over the full age range, indicating that nonlinear regression may be more suitable for depicting the relationship between lung age and spirometric parameters.

Considering the potential application value of lung age in the management of chronic respiratory diseases, this study aimed to develop new lung age estimation equations and hypothesized that nonlinear regression was a more appropriate method to build the equations. Furthermore, the lung age of patients with chronic obstructive pulmonary disease (COPD) and asthma was estimated to explore the clinical application of lung age in chronic respiratory diseases.

## Results

### Demographic characteristics

**Fig. 1:**
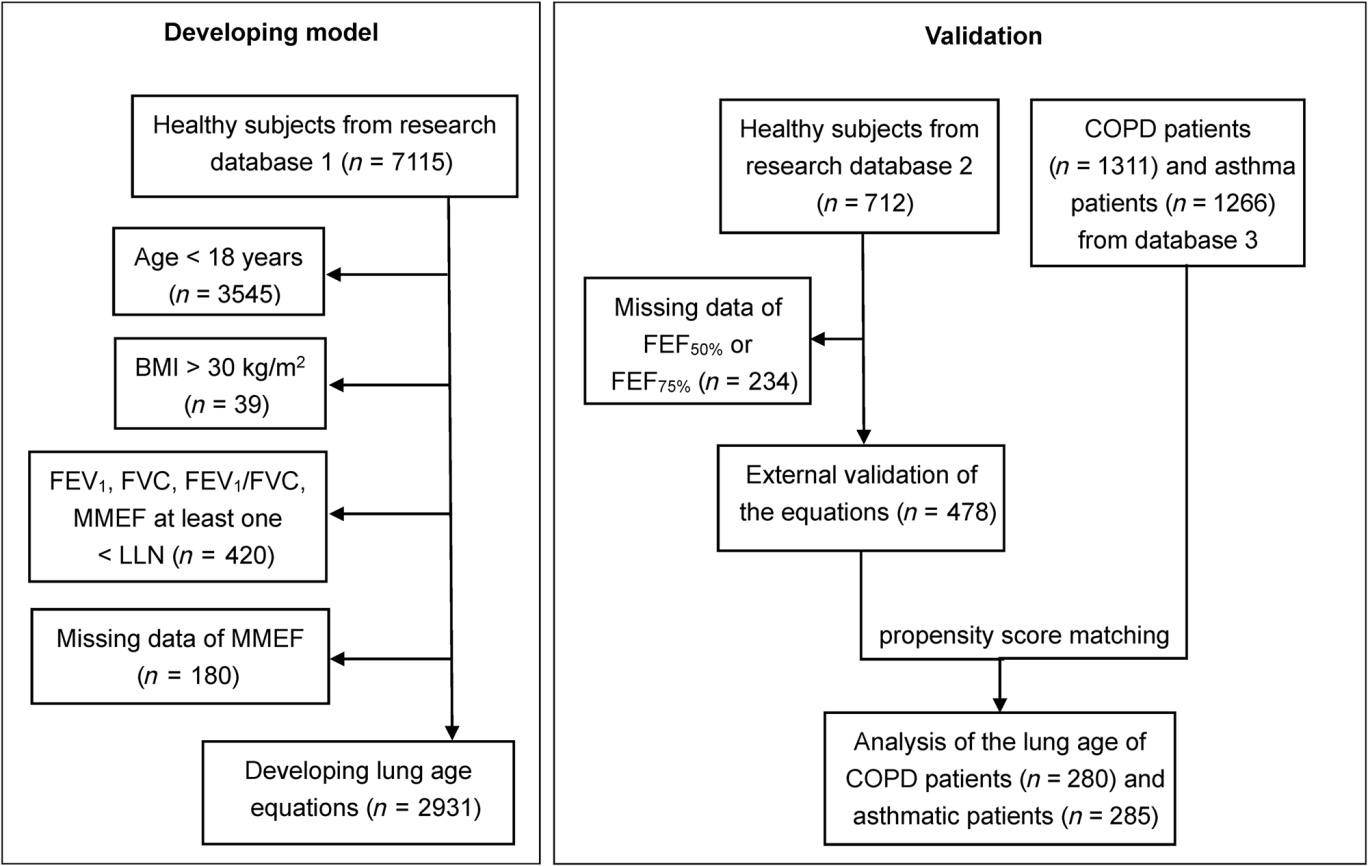
Study flow chart. The left panel displays the data inclusion and exclusion of healthy subjects in the modelling group, the right panel displays the data inclusion and exclusion of healthy subjects in the validation group and patients with COPD and asthma.

As shown in Fig. 1, 2931 healthy subjects were included in the modelling group, 478 healthy subjects were included in the validation group. The demographic characteristics and spirometric variables of the healthy subjects are presented in Table 1 and Supplementary Fig. 1. After propensity score matching (PSM), 280 patients with COPD (70 patients in each stage) and 70 COPD-matched healthy subjects were included in the analysis. As the number of stage IV asthmatic patients was limited, PSM was performed between stage I-III asthmatic patients and healthy subjects, and 285 asthmatic patients (78 patients in stage I–III, 51 patients in stage IV) and 78 asthma-matched healthy subjects were included in the analysis. The distributions of age, height and sex ratio were similar between the matched healthy subjects and patients with COPD or asthma (Tables 2 and 3).

**Table 1:** Demographic characteristics and spirometric parameters (mean ± standard deviation) of healthy subjects.

	Modelling group (*n* = 2931)	Validation group (*n* = 478)
Male/Female	1379/1552	192/286
Age, years	37.85 ± 14.92	42.41 ± 15.67
Height, cm	170.21 ± 6.06	162.53 ± 8.32
Weight, kg	67.65 ± 9.60	62.30 ± 10.80
FEV_1_, L	3.81 ± 0.63	3.11 ± 0.75
FEV_1_%Pred	101.94 ± 9.64	103.52 ± 9.53
FVC, L	4.54 ± 0.69	3.75 ± 0.88
FEV_1_/FVC	0.84 ± 0.06	0.83 ± 0.06
MMEF, L/s	4.02 ± 1.13	3.17 ± 1.06
FEF_50%_, L/s	4.83 ± 1.27	4.01 ± 1.17
FEF_75%_, L/s	1.91 ± 0.79	1.39 ± 0.70

*FEV*_*1*_ forced expiratory volume in 1 s, *FEV*_*1*_*%Pred* percentage of predicted value of FEV_1_, *FVC* forced vital capacity, *FEV*_*1*_*/FVC* ratio of FEV_1_ to FVC, *MMEF* maximum mid-expiratory flow, *FEF*_*50%*_ forced expiratory flow at 50% of FVC, *FEF*_*75%*_ forced expiratory flow at 75% of FVC.

**Table 2:** Demographic characteristics and spirometric parameters (mean ± standard deviation) of COPD patients and matched healthy subjects.

	Matched Healthy (*n* = 70)	Stage I COPD (*n* = 70)	Stage II COPD (*n* = 70)	Stage III COPD (*n* = 70)	Stage IV COPD (*n* = 70)
Male/Female	58/12	59/11	57/13	58/12	58/12
Age, years	62.16 ± 7.72	61.85 ± 7.07	62.26 ± 6.95	62.43 ± 7. 40	63.79 ± 6.93
Height, cm	162.48 ± 6.66	164.12 ± 6.96	164.28 ± 7.27	164.32 ± 6.90	163.06 ± 8.45
Weight, kg	63.78 ± 9.57	63.28 ± 10.46	60.65 ± 11.17	59.16 ± 10.54	54.71 ± 10.29
FEV_1_, L	2.83 ± 0.57	2.46 ± 0.37	1.81 ± 0.34	1.12 ± 0.25	0.66 ± 0.15
FEV_1_%Pred, %	101.25 ± 11.00	87.84 ± 5.64	65.00 ± 8.45	39.99 ± 6.31	24.26 ± 3.89
FVC, L	3.57 ± 0.67	3.87 ± 0.57	3.14 ± 0.54	2.58 ± 0.66	1.94 ± 0.46
FEV_1_/FVC	0.79 ± 0.05	0.63 ± 0.04	0.58 ± 0.06	0.44 ± 0.07	0.34 ± 0.06
MMEF, L/s	2.66 ± 0.94	1.18 ± 0.25	0.75 ± 0.26	0.40 ± 0.11	0.24 ± 0.06
FEF_50%_, L/s	3.76 ± 1.17	1.62 ± 0.52	1.02 ± 0.39	0.48 ± 0.15	0.26 ± 0.09
FEF_75%_, L/s	1.17 ± 0.51	0.39 ± 0.16	0.30 ± 0.10	0.20 ± 0.06	0.14 ± 0.05

*FEV*_*1*_ forced expiratory volume in 1 s, *FEV*_*1*_*%Pred* percentage of predicted value of FEV_1_, *FVC* forced vital capacity, *FEV*_*1*_*/FVC* ratio of FEV_1_ to FVC, *MMEF* maximum mid-expiratory flow, *FEF*_*50%*_ forced expiratory flow at 50% of FVC, *FEF*_*75%*_ forced expiratory flow at 75% of FVC.

**Table 3:** Demographic characteristics and spirometric parameters (mean ± standard deviation) of asthmatic patients and matched healthy subjects.

	Matched Healthy (*n* = 78)	Stage I Asthma (*n* = 78)	Stage II Asthma (*n* = 78)	Stage III Asthma (*n* = 78)	Stage IV Asthma (*n* = 52)
Male/Female	39/43	39/43	42/40	44/38	28/15
Age, years	50.79 ± 14.42	50.87 ± 12.51	50.23 ± 13.22	49.30 ± 15.08	55.01 ± 13.13
Height, cm	160.10 ± 8.39	161.56 ± 7.51	162.89 ± 8.29	161.19 ± 9.34	160.30 ± 7.92
Weight, kg	61.89 ± 9.57	63.69 ± 12.44	64.67 ± 11.41	61.37 ± 11.31	57.61 ± 11.27
FEV_1_, L	2.93 ± 0.80	2.73 ± 0.67	1.94 ± 0.47	1.20 ± 0.35	0.65 ± 0.17
FEV_1_%Pred, %	101.73 ± 10.44	92.39 ± 8.23	62.94 ± 7.92	40.43 ± 5.86	23.52 ± 4.72
FVC, L	3.61 ± 0.93	3.68 ± 0.89	3.27 ± 0.84	2.57 ± 0.86	1.84 ± 0.49
FEV_1_/FVC	0.81 ± 0.05	0.71 ± 0.08	0.59 ± 0.07	0.43 ± 0.08	0.35 ± 0.07
MMEF, L/s	2.89 ± 1.02	2.03 ± 0.89	1.07 ± 0.58	0.46 ± 0.39	0. 39 ± 0.63
FEF_50%_, L/s	3.84 ± 1.15	2.69 ± 1.05	1.28 ± 0.55	0.54 ± 0.20	0.34 ± 0.57
FEF_75%_, L/s	1.11 ± 0.58	0.77 ± 0.45	0.37 ± 0.19	0.19 ± 0.09	0.15 ± 0.21

*FEV*_*1*_ forced expiratory volume in 1 s, *FEV*_*1*_*%Pred* percentage of predicted value of FEV_1_, *FVC* forced vital capacity, *FEV*_*1*_*/FVC* ratio of FEV_1_ to FVC, *MMEF* maximum mid-expiratory flow, *FEF*_*50%*_ forced expiratory flow at 50% of FVC, *FEF*_*75%*_ forced expiratory flow at 75% of FVC.

### Lung age estimation equations

**Fig. 2:**
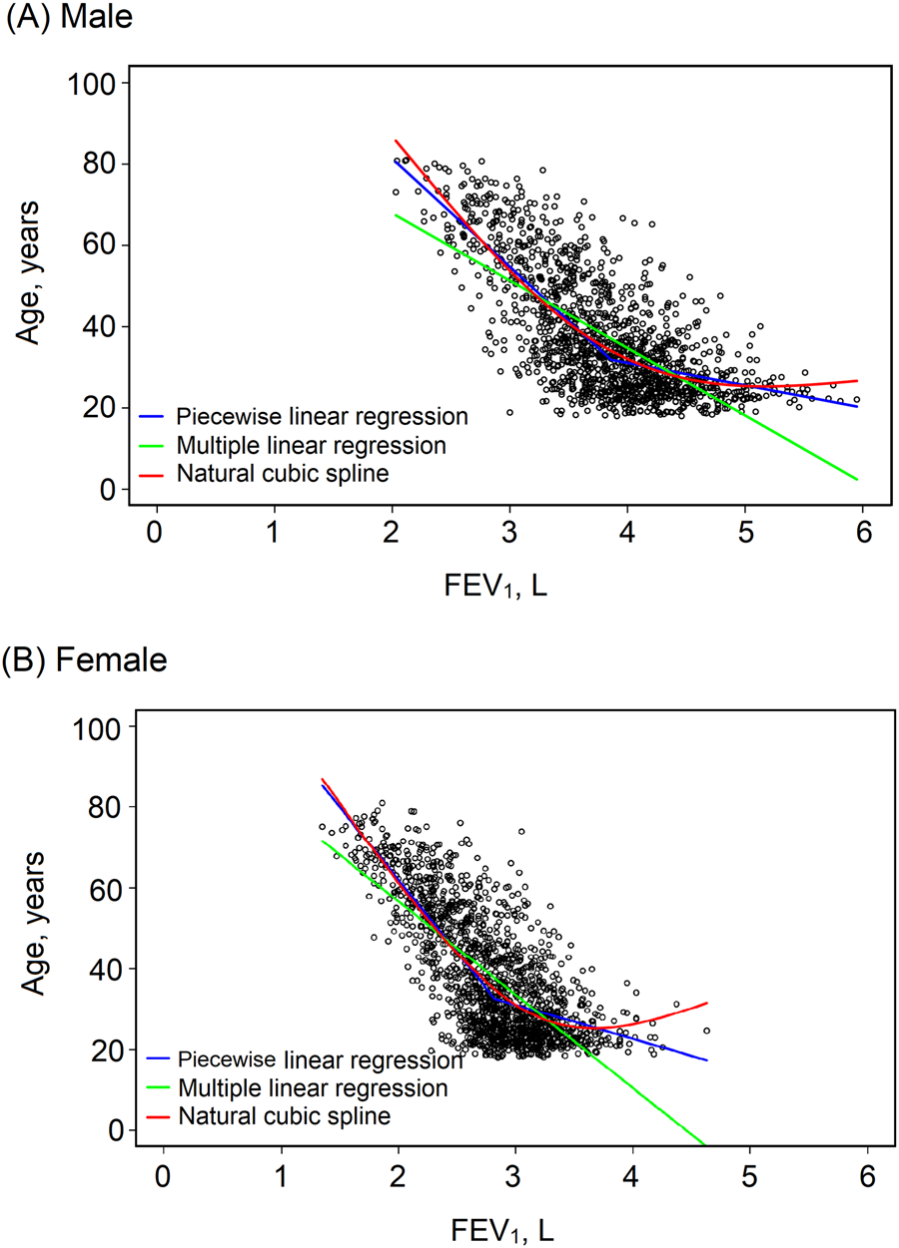
Fitting curves of the lung age estimation equations developed by different methods. Panel **A**, fitting curves of lung age estimation equatitons in males; Panel **B**, fitting curves of lung age estimation equatitons in females.

A series of models composed of different variables were built by multiple linear regression, piecewise linear regression, and the natural cubic spline method, respectively. Models with the highest adjusted *R*^2^ values of each method are presented in Fig. 2 and Supplementary Table 1. Among these models, the one with the highest adjusted *R*^2^ was built by the spline method and composed of FEV_1_, FEF_50%_, FEF_75%_, and height as explanatory variables (Table 4). This model was defined as the estimation equation of lung age and was used to derive lung age for patient groups.

**Table 4:** Lung age estimation equations developed by the spline method.

Equation	Variable	Coefficient (95% CI)	Adjusted *R*^2^	*P*	RSE
Male	Intercept	2.25 (−14.24, 18.74)	0.660	<0.001	8.693
	Height	0.49 (0.39, 0.59)			
	FEF_50%_	3.47 (2.89, 4.05)			
	FEF_75%_	−8.92 (−9.98, −7.85)			
	ns (FEV_1_)	offered in supplementary material 2			
Female	Intercept	28.49 (13.3, 43.68)	0.689	<0.001	8.544
	Height	0.36 (0.26, 0.45)			
	FEF_50%_	4.45 (3.73, 5.17)			
	FEF_75%_	−12.52 (−13.61, −11.42)			
	ns (FEV_1_)	offered in supplementary material 2			

*FEF*_*50%*_ forced expiratory flow at 50% of FVC, *FEF*_*75%*_ forced expiratory flow at 75% of FVC, *FEV*_*1*_ forced expiratory volume in 1 s, *ns (FEV*_*1*_*)* coefficient of the natural cubic spline of FEV_1_, which can be looked up in the supplementary material 2; *R*^2^ coefficient of determination, *RSE* residual standard error.

Internal validation showed that the coefficients of independent variables, adjusted *R*^2^ and mean square error (MSE) of the models of bootstrap validation were similar to those of the primary model (see Supplementary Table 2), suggesting that the equations developed in this study performed well in internal prediction. External validation showed that the MSE of ∆ lung age in the validation group (69.9) was smaller than that of the modelling group (73.0–75.6), indicating that the validation group did not present larger differences between the estimated lung age and the chronological age compared to the modelling group (Supplementary Tables 2 and 3). The MSE of ∆ lung age estimated by nonlinear regression (piecewise linear regression: 69.3, spline method: 69.9) was smaller than that by the multiple linear regression (78.8), suggesting nonlinear regression had smaller errors than the multiple linear regression in estimating lung age in validation group (Supplementary Table 3).

### Upper normal limit of ∆ lung age

Lung age and ∆ lung age of healthy subjects in the modelling group were calculated using the new lung age equations. As ∆ lung age is of greater practical use in the interpretation of lung age, the normal limit of ∆ lung age was explored. Analysis of the ∆ lung age of the modelling group showed that ∆ lung age was negatively correlated with chronological age but not with height or FEV_1_ (shown in Supplementary Fig. 2), and there was no significant difference in ∆ lung age between healthy males and females (male median: 0.92 years, female median: 0.89 years, *P* = 0.966). Thus, age-dependent normal limits of ∆ lung age were derived from a regression model between ∆ lung age and chronological age (Supplementary Fig. 2a). Since those with higher ∆ lung age (older lung age) are of greater clinical interest, we only derived the upper limit of normal (ULN) of ∆ lung age, which was calculated according to the results of the regression model as follows: ULN of ∆ lung age (years) = 12.243–0.323 × Age (years) + 1.645 × 7.037 (residual standard error). In addition, we derived a constant ULN of ∆ lung age by calculating the 95th percentile of the healthy subjects, which was 12.5 years. To compare the practical use of lung age estimated by different regression methods, we also derived the ULN of ∆ lung age estimated by the multiple linear regression (MLR) method in the same way, that is, the ULN of ∆ lung age (MLR) (years) = 14.690–0.392 × Age (years) + 1.645 × 7.387.

### Proportion of subjects with ∆ lung age above the ULN

**Fig. 3:**
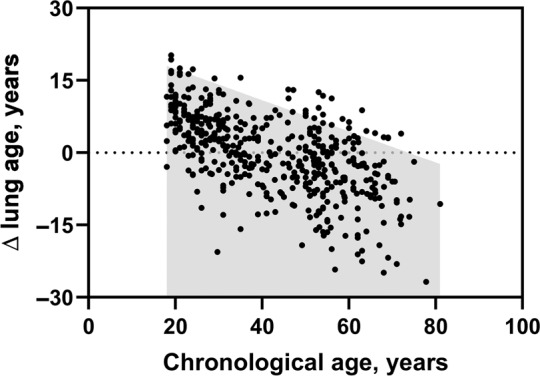
∆ lung age of healthy subjects in the validation group. The grey area represents the upper limit of normal of the ∆ lung age.

As shown in Table 5, the proportions of patients with ∆ lung age above different ULNs (age-dependent ULN derived by spline method, constant ULN derived by spline method, age-dependent ULN derived by multiple linear regression, ULN proposed by the previous study [Yamaguchi et al., 2012]^11^) were compared. The age-dependent ULN derived by the spline method identified more patients with COPD or asthma than other ULNs, with 52.9% of stage I and 100% of stage II-IV COPD patients exceeding the age-dependent ULN (Table 5). For healthy subjects in the validation group, 94.1% (450/478) of ∆ lung age was within the age-dependent ULN derived by spline method (Fig. 3), indicating the equations and the derived normal limit are acceptable in healthy subjects.

**Table 5:** Number (percentage) of patients with ∆ lung age over the ULN.

	Stage I		Stage II	Stage III	Stage IV
Patients with COPD					
Age-dependent ULN		37/70 (52.9%)	70/70 (100.0%)	70/70 (100.0%)	70/70 (100.0%)
Constant ULN		9/70 (12.8%)	67/70 (95.7%)	70/70 (100.0%)	70/70 (100.0%)
Age-dependent ULN-MLR		1/70 (1.4%)	48/70 (68.6%)	70/70 (100.0%)	70/70 (100.0%)
ULN-Yamaguchi et al. [11]		14/70 (20.0%)	47/70 (67.1%)	69/70 (98.6%)	70/70 (100.0%)
Patients with asthma					
Age-dependent ULN		20/78 (25.6%)	77/78 (98.7%)	78/78 (100.0%)	51/51 (100.0%)
Constant ULN		11/78 (13.9%)	70/78 (88.6%)	78/78 (100.0%)	51/51 (100.0%)
Age-dependent ULN-MLR		6/78 (7.7%)	63/78 (80.7%)	78/78 (100.0%)	51/51 (100.0%)
ULN-Yamaguchi et al. [11]		23/78 (29.5%)	49/78 (62.8%)	76/78 (97.5%)	51/51 (100.0%)

*ULN* upper limit of normal; age-dependent ULN, age-dependent ULN of ∆ lung age estimated by the spline method; constant ULN, 95th percentile of the healthy subjects of ∆ lung age estimated by the spline method (12.5 years); ULN-MLR, age-dependent ULN of ∆ lung age estimated by multiple linear regression; ULN-Yamaguchi et al., ULN of ∆ lung age proposed by the previous study of Yamaguchi et al. (13.4 years in males and 15.0 years in females)^11^.

### ∆ lung age of COPD and asthma

**Fig. 4:**
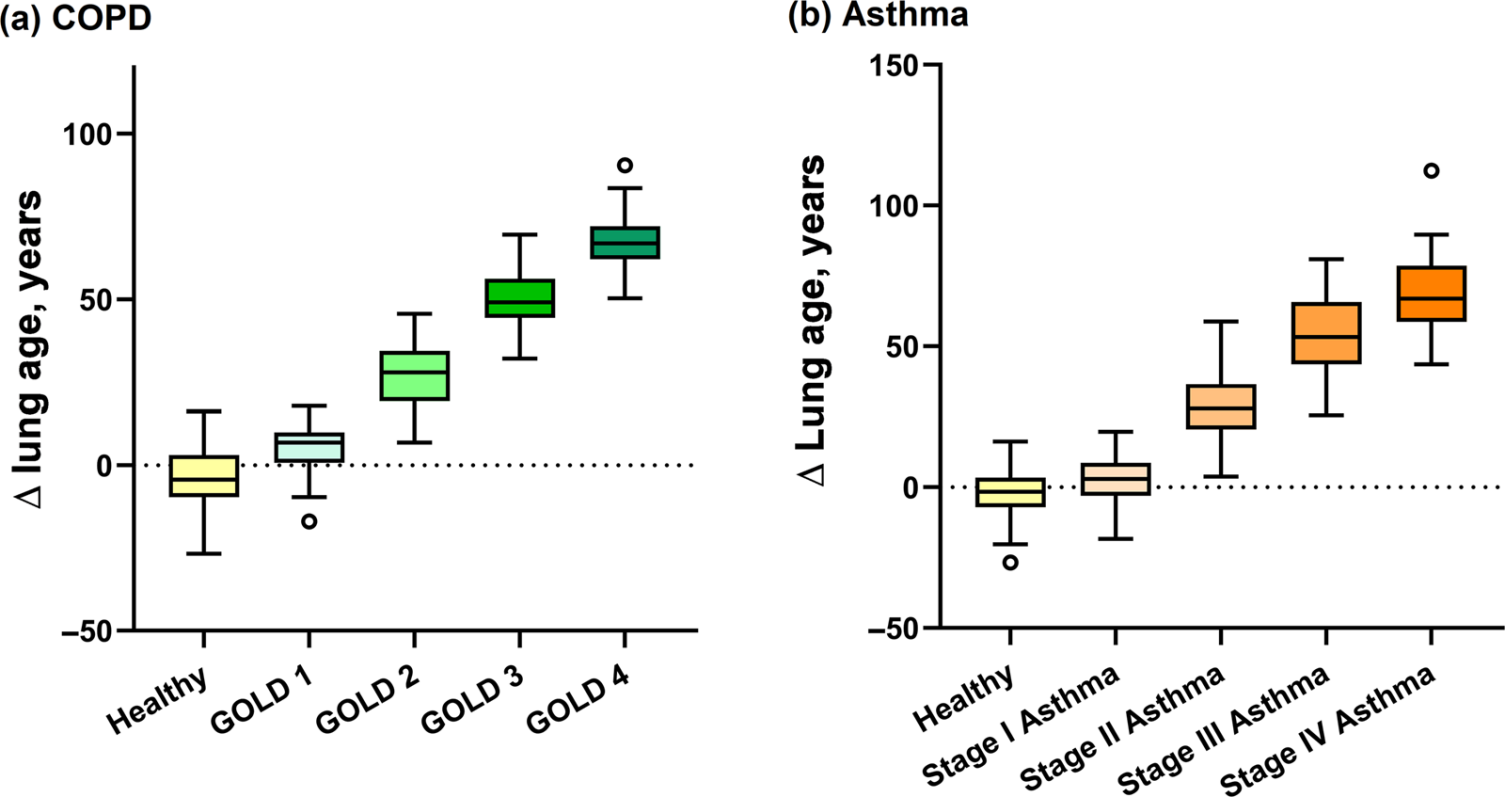
Comparisons of ∆ lung age between matched healthy subjects and patients with COPD and asthma. Stage I, 80% ≤FEV_1_ %pred; Stage II, 50% ≤FEV_1_ %pred <80%; Stage III, 30% ≤FEV_1_ %pred <50%; Stage IV, 30% >FEV_1_ %pred. The centre line in the box indicates the median, the lower and upper bound of the box and whiskers indicate the first and the third quartile, and the minimum and maximum value, the point indicates the outlier.

As shown in Fig. 4, the ∆ lung age of stage I COPD patients (Mean ± SD: 4.88 ± 6.81 years) was higher than that of the matched healthy subjects (−4.59 ± 9.42 years, *P* < 0.05), and a progressive increase in ∆ lung age was shown in stage I–IV COPD patients (stage II: 25.85 ± 9.30 years, stage III: 50.56 ± 9.00 years, stage IV: 65.43 ± 10.08 years, between-group *P* < 0.05). Similarly, the ∆ lung age of stage I asthmatic patients (2.45 ± 9.16 years) was higher than that of the matched healthy subjects (−1.95 ± 7.99 years, *P* < 0.05), and a progressive increase in ∆ lung age was shown in stage I–IV COPD patients (stage II: 28.44 ± 11.63 years, stage III: 54.27 ± 12.90 years, stage IV: 68.85 ± 12.77 years, between-group *P* < 0.05).

## Discussion

In this study, spirometry-derived lung age estimation equations were developed based on the data of healthy nonsmokers aged 18–80 years old by a spline method. Analysis of lung age in patients with COPD and asthma revealed that ∆ lung age progressively increases with the degree of airflow limitation.

Scatter plots of spirometric parameters against age in healthy subjects of the modelling group (Fig. 2) demonstrated a similar result as previous studies^13,14^ that the relationship between age and spirometric parameters is nonlinear. The present study showed that building lung age equations by using nonlinear regression (piecewise linear regression or spline method) could improve the goodness of fit of the equations and had smaller errors in estimating lung age compared with multiple linear regression. Moreover, lung age estimated by the nonlinear regression method could identify more patients with COPD or asthma than that estimated by linear regression. These findings confirm our hypothesis that nonlinear regression is more suitable for developing estimation equations of lung age. Though the lung age values estimated by the piecewise linear regression and the spline method seemed to be similar in Fig. 2, we chose the equations built by the spline method as the lung age equations given its higher adjusted *R*^2^ and the continuity of the equations. Calculation tools for the estimation of lung age with our new equations are available at https://cltshiny.shinyapps.io/LungAge/ for an individual and in an Excel spreadsheet in the Supplementary material for large datasets.

While several studies have developed equations for the estimation of lung age^15^, the interpretation of lung age remains controversial. When first proposed, lung age was used to reflect the functional damage or premature aging of the lungs caused by smoking^4,9^. However, Quanjer et al.^8^ questioned whether an older lung age (a mean value estimated by the backward calculation method) should be interpreted as lung damage caused by smoking, as it disregarded the variability between individuals. Yamaguchi et al.^11^ and Ben Saad et al.^16^ proposed using a three-step procedure based on the limits of normal to judge the abnormality of lung age: if the calculated lung age of an individual is within the ULN and LLN (lower limit of normal), then his or her lung age should be interpreted to be consistent with his or her chronological age, otherwise, his or her lung age is judged to be older (when lung age >ULN) or younger (when lung age <LLN) than the chronological age. Based on their study populations, the LLN/ULN of ∆ lung age proposed by Yamaguchi et al.^11^ or Ben Saad et al.^16^ was −13.4/+13.4 years or −16.90/+16.90 years in males, and −15.0/+15.0 years or −14.77/+14.77 years in females, respectively. Our study showed that the ∆ lung age of healthy subjects was correlated with chronological age, and the age-dependent ULN of ∆ lung age displayed a better performance than the constant ULN in identifying patients with diseases, especially for mild patients. Thus, we propose to use an age-dependent normal limit of ∆ lung age instead of a constant normal limit in the interpretation of lung age. As demonstrated in Table 5, ULN derived by our new method could markedly improve the capacity to identify patients with COPD and asthma when compared with the ULN of the previous study (Yamaguchi et al. 2012^11^). Different from previous studies, we also suggest that estimated lung age, instead of the chronological age, should be adopted when the lung age value is above the chronological age but within the ULN, so that such individuals can be aware of their lower lung function compared to that of the population and thus be more active in early intervention, such as smoking cessation.

To our knowledge, this is the first study to analyze the levels of ∆ lung age in COPD and asthmatic patients with varying airflow limitations. Although FEV_1_ or FEV_1_%pred is the “gold standard” in the assessment of lung function in clinical or research fields^2^, it may be abstract for some patients to understand. In this study, we found that ∆ lung age progressively increases with the degree of airflow limitation, suggesting that lung age may be used as a simple surrogate to inform patients about the severity of their disease. Moreover, even though the FEV_1_ of stage I COPD patients was numerically normal (FEV_1_%pred ≥80%), the ∆ lung age values of 53% stage I COPD patients exceeded the ULN, indicating that lung age may be more helpful than FEV_1_%pred for patients to be aware of their lung function impairment. As lung age makes it easier for patients to understand their lung function level and is also well accepted by the majority of primary care physicians^17^, we believe that lung age may be a useful tool to be applied in the assessment and management of chronic respiratory diseases with lung function impairment, especially in primary care.

Aiming at developing lung age equations based on the data of healthy subjects, the present study excluded smokers from the analysis. It may be of more practical value to include smokers or those with occupational exposures for analyzing the ∆ lung age of those at risk of diseases. What’s more, the analysis of ∆ lung age of disease patients was exploratory, and we did not perform sample size estimation with a prior hypothesis, thus, though the between-group *P*-value was <0.05, it did not promise the between-group difference was clinically significant. The practical application value of lung age in chronic respiratory diseases should be validated in prospective studies.

In conclusion, spirometry-derived lung age estimation equations were developed by a spline modelling method. ∆ Lung age derived by the new estimation equations can reflect the level of lung function in patients with COPD and asthma. Thus, lung age may be used in the assessment of chronic obstructive respiratory diseases by both health care providers and patients to better understand and manage the disease.

## Methods

The study was based on retrospective data obtained from research databases. Firstly, spirometric lung age estimation equations were established and validated based on the data of healthy nonsmokers. Secondly, lung age and ∆ lung age of patients with COPD and asthma were analyzed. The study was performed in accordance with the Declaration of Helsinki and was approved by the Ethics Committee of First Affiliated Hospital of Guangzhou Medical University with a waiver of informed consent as it is a retrospective study (approval number: No. 2019-72).

### Study population

Healthy subjects of this study included the modelling group and the validation group, the former was used for the establishment and internal validation of the lung age equations, and the latter was used for the external validation. Subjects of the modelling group were from the “Reference Values for Spirometry in Chinese Aged 4–80 Years” study that collected multicenter spirometric data in 2007–2010 (research database 1). Subjects of the validation group were from the “Reference Values for Respiratory Impedance with Impulse Oscillometry in Healthy Chinese Adults” study that collected multicenter spirometric data in 2016–2018 (research database 2). Details of these studies have been reported previously^14,18^. Briefly, the inclusion criteria of healthy subjects in this study were: age 18–80 years old; no history of smoking or occupational exposures; no symptoms or history of chronic cardiopulmonary diseases; body mass index ≤ 30 kg/m^2^; and FEV_1_, forced vital capacity (FVC), FEV_1_/FVC, and maximum mid-expiratory flow (MMEF) all within the normal limits.

Spirometric data and medical records of patients with COPD or asthma from 2016 to 2019 were derived from the Respiratory Health Big Data database of Guangzhou Respiratory Health Institute, First Affiliated Hospital of Guangzhou Medical University (database 3). Patients with COPD included in the analysis met the following criteria: clinically diagnosed as COPD according to the guideline of the Global Initiative for Chronic Obstructive Lung Disease (GOLD)^2^; ≥40 years old; no exacerbation within the last 4 weeks before the spirometry measurement; and no history of asthma, interstitial lung diseases, pulmonary tuberculosis, or lung cancer. Patients with asthma included in the analysis met the following criteria: clinically diagnosed as asthma according to the guideline of the Global Initiative for Asthma^19^; ≥18 years old; and no history of COPD, interstitial lung diseases, pulmonary tuberculosis, or lung cancer. The severity of airflow limitation in patients with COPD was categorized according to GOLD^2^: stage I (80% ≤FEV_1_ %pred), stage II (50% ≤FEV_1_ %pred <80%), stage III (30% ≤FEV_1_ %pred <50%) and stage IV (30% <FEV1 %pred). For comparability, patients with asthma were also categorized according to the same criteria.

### Spirometric parameters

Spirometric parameters analyzed in this study included FEV_1_, FVC, FEV_1_/FVC, MMEF, FEF_50%_ (forced expiratory flow at 50% of FVC), and FEF_75%_ (forced expiratory flow at 75% of FVC).

### Statistical analysis

One-way ANOVA was used for the comparisons of age, height, weight and ∆ lung age of multiple groups, and Tamhane’s T2 test was used for post hoc multiple comparisons as the variances were unequal. The chi-square test was used for the comparisons of sex ratio.

Sex-specific lung age estimation models with the chronological age of healthy subjects as the dependent variable and spirometric parameters and height as explanatory variables were built via multiple linear regression, piecewise linear regression, and the natural cubic spline method. The goodness of fit of the model was assessed by the adjusted coefficient of determination (*R*^2^). The model with the highest adjusted *R*^2^ was used as the final estimation equations of lung age. The final model was internally validated using the bootstrap resampling method. Differences between the estimated lung age and the chronological age (∆ lung age) of healthy subjects in the validation group, and the proportion of ∆ lung age exceeding the normal limit in the validation group were analyzed for the external validation of the equations.

As the distributions of age and height, and the proportion of sex were different between healthy subjects and patients with COPD or asthma, PSM was performed to balance these factors between healthy subjects and patients. R^®^ Version 4.0.3 and GraphPad Prism^®^ Version 8.0.1 were used in the analysis and for graphics.

### Reporting summary

Further information on research design is available in the Nature Research Reporting Summary linked to this article.

## Supplementary information

Supplementary Information

Supplementary Date

Reporting Summary

## Data Availability

The datasets used and/or analyzed during the current study are available from the corresponding author (Dr. Jinping Zheng, jpzhenggy@163.com).

## References

[1] Abbafati C (2020). Global burden of 369 diseases and injuries in 204 countries and territories, 1990–2019: a systematic analysis for the Global Burden of Disease Study 2019. Lancet.

[2] Global Initiative for Chronic Obstructive Lung Disease. Global Strategy for the Diagnosis, Management, and Prevention of Chronic Obstructive Pulmonary Disease. http://www.goldcopd.org (2020).

[3] Graham BL (2019). Standardization of spirometry 2019 update an official American Thoracic Society and European Respiratory Society technical statement. Am. J. Respiratory Crit. Care Med..

[4] Morris JF, Temple W (1985). Spirometric ‘lung age’ estimation for motivating smoking cessation. Prev. Med. (Balt.)..

[5] Parkes G, Greenhalgh T, Griffin M, Dent R (2008). Effect on smoking quit rate of telling patients their lung age: The Step2quit randomised controlled trial. Bmj.

[6] Peixoto-Souza FS (2013). Lung age in women with morbid obesity. Rev. Assoc. Med. Bras..

[7] Carr WW, McDonald M, Meizlik P (2019). Effect of intravenously administered reslizumab on spirometric lung age in patients with moderate-to-severe eosinophilic asthma. Allergy Asthma Proc..

[8] Quanjer PH, Enright PL (2010). Should we use ‘lung age’?. Prim. Care Respiratory J..

[9] Hansen JE, Wasserman K, Sun XG, Wasserman K (2010). Calculating gambling odds and lung ages for smokers. Eur. Respir. J..

[10] Newbury W, Newbury J, Briggs N, Crockett A (2010). Exploring the need to update lung age equations. Prim. Care Respir. J..

[11] Yamaguchi K (2012). Novel regression equations predicting lung age from varied spirometric parameters. Respir. Physiol. Neurobiol..

[12] Yamaguchi K, Onizawa S, Tsuji T, Aoshiba K, Nagai A (2011). How to evaluate ‘Spirometric’ lung age - What method is approvable?. Respir. Physiol. Neurobiol..

[13] Quanjer PH (2012). Multi-ethnic reference values for spirometry for the 3-95-yr age range: The global lung function 2012 equations. Eur. Respir. J..

[14] Jian W (2017). Reference values for spirometry in Chinese aged 4–80 years. J. Thorac. Dis..

[15] Khelifa MB (2018). “Spirometric” lung age reference equations: A narrative review. Respiratory Physiol. Neurobiol..

[16] Saad HB (2013). Estimated lung age in healthy North African adults cannot be predicted using reference equations derived from other populations. Egypt. J. Chest Dis. Tuberc..

[17] Parker DR (2015). Primary Care Providers’ Views on Using Lung Age as an Aid to Smoking Cessation Counseling for Patients with Chronic Obstructive Pulmonary Disease. Lung.

[18] Liang XL (2021). Reference values of respiratory impedance with impulse oscillometry in healthy Chinese adults. J. Thorac. Dis..

[19] Global Initiative for Asthma. Global Strategy for Asthma Management and Prevention. http://www.ginasthma.org (2020).

