# Supplementary material for: Estimation of lung age via a spline method and its application in chronic respiratory diseases
Source: NPJ Prim Care Respir Med. 2022 Sep 29;32:36. doi: 10.1038/s41533-022-00293-9 (PMC9522795; doi:10.1038/s41533-022-00293-9)
Supplement: Supplementary file 1 — Supplementary Information [file 41533_2022_293_MOESM1_ESM.pdf]

SUPPLEMENTARY INFORMATION

Table S1 Models with the highest adjusted R^2 (coefficient of determination) for each method.

Regression analysis	Model		R^2	Adjusted R^2	P	RSE
**Multiple linear regression**	M	LA =8.30 +0.53 ×H -15.67 ×FEV_1 +3.24 ×$FEF_{50\%}$ -8.60 ×$FEF_{75\%}$.	0.590	0.589	<0.001	9.556
	F	LA =28.55 +0.40 ×H -18.47 ×FEV_1 +4.06 ×$FEF_{50\%}$ -11.84 ×$FEF_{75\%}$.	0.624	0.623	<0.001	9.410
**Piecewise linear regression**	M	FEV_1 ≤3.67 L: LA =56.11 +0.48 ×H -28.09 ×FEV_1 +3.44 ×$FEF_{50\%}$ -8.91 ×$FEF_{75\%}$.	0.637	0.636	<0.001	8.991
		FEV_1 >3.67 L: LA = -21.77 +0.48×H -6.84×FEV_1 + 3.44×$FEF_{50\%}$ -8.91×$FEF_{75\%}$.				
	F	FEV_1 ≤2.81 L: LA =68.55 +0.34 ×H -31.27 ×FEV_1 +4.25 ×$FEF_{50\%}$ -12.18 ×$FEF_{75\%}$.	0.682	0.681	<0.001	8.648
		FEV_1 >2.81 L: LA = -9.17 +0.34 ×H -3.56 ×FEV_1 +4.25 ×$FEF_{50\%}$ -12.18 ×$FEF_{75\%}$.				
**Natural cubic spline method**	M	LA =2.25 +0.49 ×H +ns (FEV_1) +3.47 ×$FEF_{50\%}$ -8.92 ×$FEF_{75\%}$	0.661	0.660	<0.001	8.693
	F	LA =28.49 +0.36 ×H+ ns (FEV_1) +4.45 ×$FEF_{50\%}$ -12.52×$FEF_{75\%}$	0.690	0.689	<0.001	8.544

F, female; H, height; LA, lung age; M, male; ns (FEV_1), coefficient of natural cubic spline of FEV_1, which was offered in the Supplementary material 2; R^2, coefficient of determination; RSE, residual standard error; FEV_1, forced expiratory volume in 1 s; $FEF_{50\%}$ forced expiratory flow at 50% of FVC; $FEF_{75\%}$, forced expiratory flow at 75% of FVC.

Table S2 Comparisons of the results between the primary model and the bootstrap validation model.

	**Primary model**	**Bootstrap validation model (95 CI%)**
**Male**		
β_{H}	0.492	0.492 (0.389, 0.595)
$\beta_{ns(FEV1)1}$	-43.812	-43.210 (-47.016, -39.405)
$\beta_{ns(FEV1)2}$	-98.074	-97.099 (-106.681, -87.517)
$\beta_{ns(FEV1)3}$	-38.070	-37.759 (-43.002, -32.517)
$\beta_{FEF50\%}$	3.470	3.489 (2.867, 4.11)
$\beta_{FEF75\%}$	-8.918	-8.933 (-10.102, -7.764)
R^2	0.661	0.663 (0.631, 0.694)
Adjusted R^2	0.660	0.661 (0.629, 0.693)
MSE	75.568	75.138 (68.959, 81.318)
**Female**		
β_{H}	0.356	0.356 (0.259, 0.453)
$\beta_{ns(FEV1)1}$	-43.058	-41.700 (-46.181, -37.219)
$\beta_{ns(FEV1)2}$	-86.060	-83.855 (-94.615, -73.096)
$\beta_{ns(FEV1)3}$	-24.562	-25.018 (-32.365, -17.671)
$\beta_{FEF50\%}$	4.452	4.441 (3.739, 5.143)
$\beta_{FEF75\%}$	-12.517	-12.524 (-13.789, -11.26)
R^2	0.690	0.692 (0.667, 0.717)
Adjusted R^2	0.689	0.690 (0.665, 0.715)
MSE	73.000	72.587 (67.158, 78.016)

β_H, coefficient of height; $\beta_{ns(FEV1)}$, natural cubic spline coefficient of FEV_1; $\beta_{FEF50\%}$, coefficient of $FEF_{50\%}$; $\beta_{FEF75\%}$, coefficient of $FEF_{75\%}$, R^2, coefficient of determination; Adjusted R^2, adjusted coefficient of determination; MSE, mean square error. 95% CI, 95% confidence interval.

Table S3 Differences between the estimated lung age and the chronological age (Δ lung age) in healthy subjects of the validation group

	Multiple linear regression	Piecewise linear regression	Natural cubic spline method
Sample size	478	478	478
Mean	-0.96	-0.48	-0.13
Median	-1.61	0.33	0.14
Min, Max	-25.52, 22.72	-26.52, 20.15	-26.77, 20.25
MSE	78.76	69.26	69.91

Min, minimum; Max, maximum; MSE, mean square error.

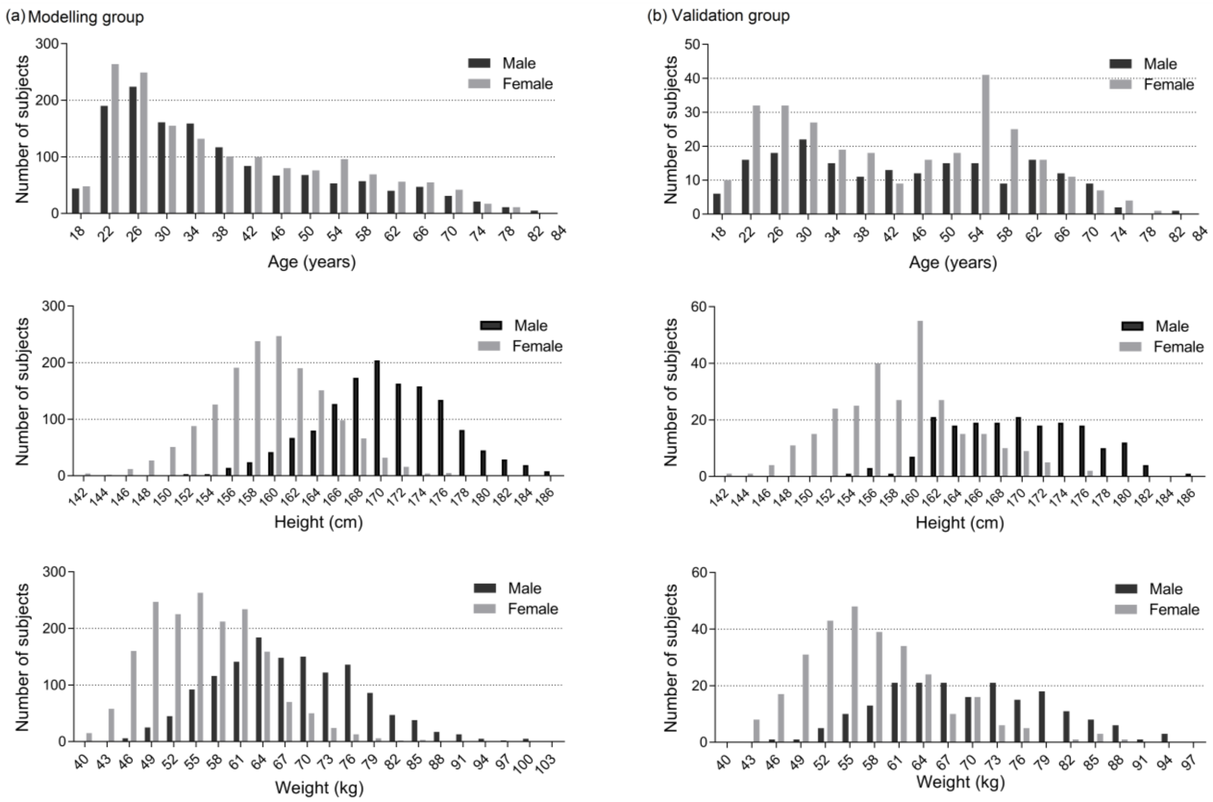

Figure S1 Distribution of age, height and weight of the healthy subjects.

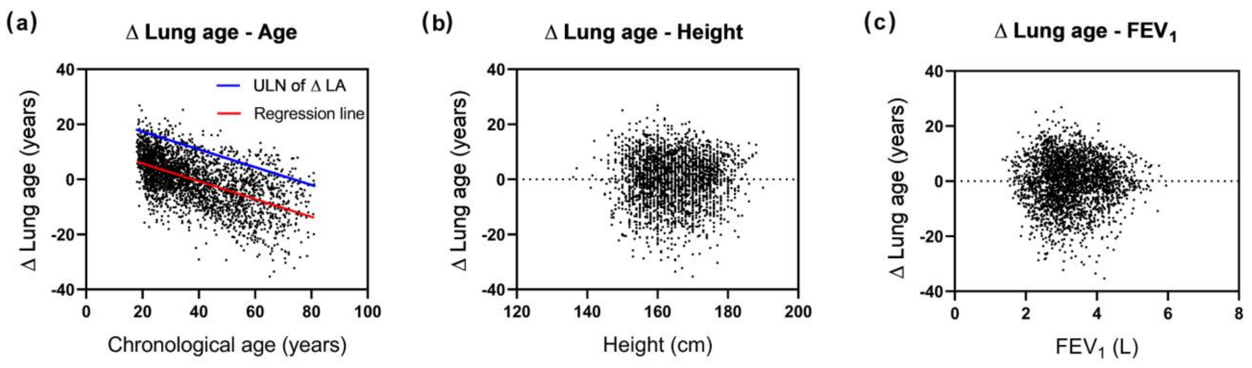

Figure S2 Scatter plots between Δ lung age and age, height and FEV_1 in healthy subjects of the modelling group.

Δ Lung age of healthy subjects was correlated with chronological age (a) but not with height (b) or FEV_1 (c). The red line in Panel A indicates the regression model between Δ lung age and chronological age (Δ lung age =12.243 [95% CI, 11.55-12.94] - 0.323 [95% CI, 0.306-0.340] × Age, R^2=0.323, P<0.01, residual standard error =7.037), and the blue line indicates the upper limit of normal (ULN) of Δ lung age derived by the regression model.

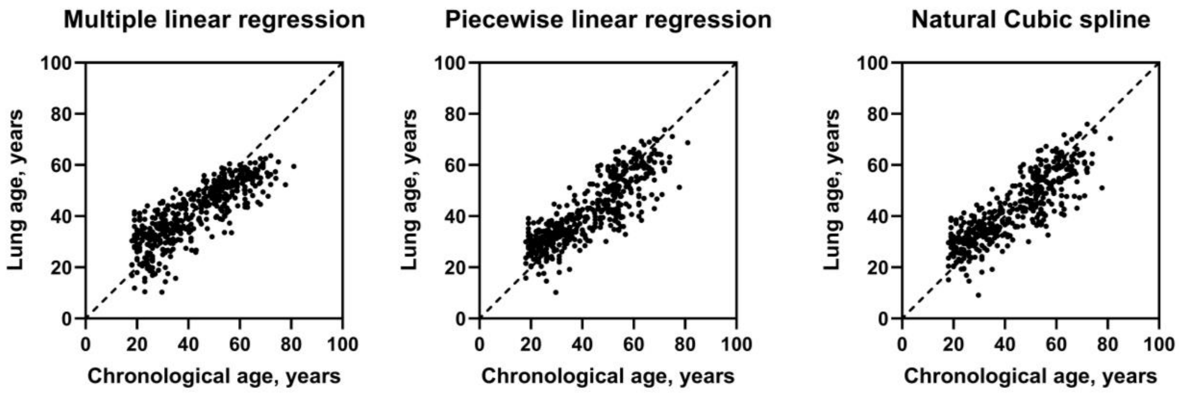

Figure S3 Scatter plots between the estimated lung age and the chronological age in healthy subjects of the validation group.

The dashed line represented the identity line, which has a slope of 1 and an intercept of 0.

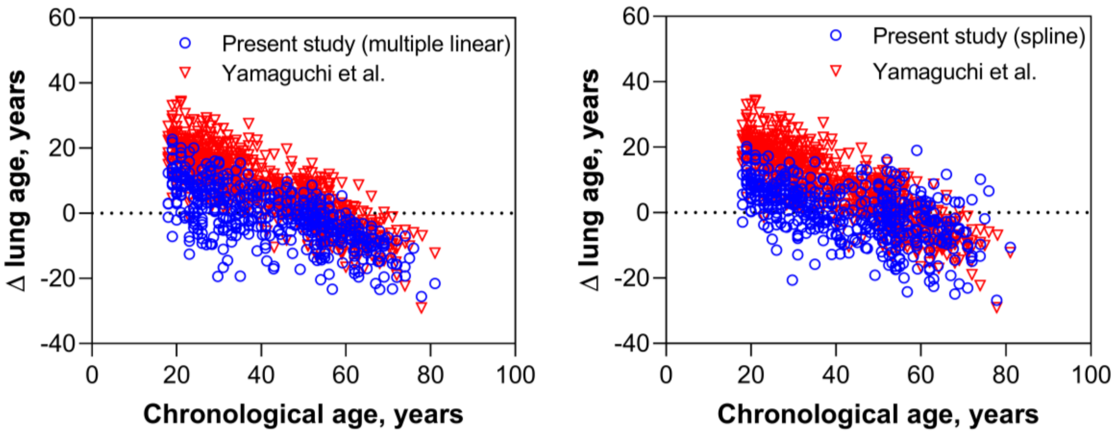

Figure S4 Comparisons on the estimated Δ lung age between the present study and the study of Yamaguchi et al. in healthy subjects of the validation group.
